# Effects of Tai Chi combined with intermediate frequency therapy on patients with chronic nonspecific neck pain: a randomized controlled trial

**DOI:** 10.3389/fpain.2025.1700212

**Published:** 2025-11-03

**Authors:** Kangni Deng, Yuheng Zhou, Jiasi Qian, Lilin Wang, Fan Yu, Bo Wang

**Affiliations:** ^1^ Department of Physical Therapy and Rehabilitation, Kunshan Sixth People’s Hospital, Jiangsu, China; ^2^ School of Exercise and Health, Shanghai University of Sport, Shanghai, China; ^3^ Department of Pain Management, Huadong Hospital, Fudan University, Shanghai, China

**Keywords:** Tai Chi, intermediate frequency, nonspecific neck pain, exercise therapy, effect

## Abstract

**Background:**

Chronic non-specific neck pain (CNSNP) is the most common type of chronic neck pain encountered in clinical practice. Existing studies have demonstrated that intermediate frequency therapy can effectively alleviate neck pain symptoms. Among other conservative treatment modalities, Tai Chi, a typical mind-body exercise, may improve musculoskeletal function and postural control, but its effect on cervical stability and CNSNP remains unclear. The aim of this study was to compare the clinical efficacy of Tai Chi combined with intermediate frequency therapy vs. intermediate frequency therapy alone in patients with CNSNP.

**Methods:**

According to the inclusion and exclusion criteria, patients with CNSNP were recruited from the rehabilitation medicine clinic of the Sixth People's Hospital of Kunshan City, resulting in the enrollment of 60 eligible participants. Patients were randomly assigned to either the experimental group (EG) or the control group (CG). The EG received Tai Chi combined with intermediate frequency therapy, while the CG received intermediate frequency therapy alone. The primary outcome was the visual analogue scale (VAS) for pain. Secondary outcomes included the Neck Disability Index (NDI), the D value of cervical physiological curvature measured by x-ray, and the cervical range of motion (ROM) score. The intervention lasted eight weeks, with sessions conducted five times per week, for a total of 40 sessions. Assessments were performed at baseline, at four weeks (mid-intervention), and at the end of eight weeks.

**Results:**

During the study, one participant in the EG withdrew after missing one week of Tai Chi intervention. Two participants in the CG discontinued: one due to a change in their treatment plan, and one for personal reasons. Thus, 57 patients with CNSNP completed the study. Both groups showed significant improvements in VAS, NDI, cervical physiological curvature (D value), and ROM scores after treatment compared to baseline. Notably, the improvement in the D value was significantly greater in the EG than in the CG.

**Conclusion:**

For patients with CNSNP, the combination of Tai Chi and intermediate frequency therapy appeared to alleviate pain and improve function. Compared to intermediate frequency therapy alone, this combined approach significantly improves the physiological curvature of the cervical spine in individuals with CNSNP. Furthermore, these findings suggest that Tai Chi may be a safe and beneficial adjunctive therapy, and may represent a promising alternative for the management of CNSNP. However, larger-scale long-term studies are still needed.

**Clinical Trial Registration:** www. itmctr.ccebtcm.org.cn, identifier (TTM-CTR-2025000447).

## 1 Introduction

In recent years, chronic neck pain (CNP) has been recognized as the fourth most common musculoskeletal disorder leading to disability (1). The most prevalent subtype of CNP is chronic nonspecific neck pain (CNSNP) (2, 3). CNSNP is defined as neck and shoulder pain that cannot be attributed to trauma, tumor, infection, or pathology of other regions (4), and is primarily associated with poor posture, mechanical stress, and degenerative changes (5). CNSNP is characterized by recurrent episodes, often resulting in a vicious cycle of chronic pain (pain persisting for more than 3 months) (6). Additionally, non-specific neck pain (NNP) affects approximately 30%–50% of the general population and frequently leads to significant disability (7).

Intermediate frequency therapy has been proven to be an effective modality for treating neck pain. By delivering isovolumic alternating currents of different frequencies into the body, this therapy stimulates muscle fibers and endogenous opioid peptide neurons in the brain, thereby enhancing blood circulation, alleviating pain, and promoting recovery of neck function (8, 9). Research indicates that while medium frequency therapy can provide short-term pain relief, its long-term efficacy depends on combination with other treatments (such as exercise therapy) to improve cervical spine function and alleviate patients' pain symptoms (8).

Current international clinical guidelines recommend conservative management as the primary treatment option for CNSNP, including exercise therapy, psychological interventions, comfort care, and health education. Among these, exercise therapy is typically regarded as the cornerstone of management (10). Exercise therapy has been shown to effectively improve short-term pain and disability in patients with CNSNP (11). Common exercise modalities include aerobic exercise, resistance training, and mind-body practices (12). Tai Chi, originating in China, is a low-intensity mind-body exercise that integrates physical movement, breathing, and meditative practices (13). Previous research indicates that Tai Chi may enhance cervical muscle stability and correct abnormal cervical curvature, thereby improving muscle strength and tension (14). Based on these observations, it is reasonable to hypothesize that Tai Chi may benefit patients with CNSNP. Moreover, the combination of Tai Chi and medium frequency therapy can transcend the limitations of single-modality treatment, offering greater benefits for patients with CNSNP. Therefore, the aim of this study was to evaluate the efficacy of Tai Chi combined with intermediate frequency therapy in patients with CNSNP, assessed through measures of pain, cervical joint mobility (ROM), x-ray evaluation of cervical physiological curvature, and neck function.

## 2 Methods

### 2.1 Study design

A single-blind, parallel-group trial with an 8-week follow-up period was conducted from March 2025 to July 2025. The study adhered to the principles of the Declaration of Helsinki and received approval from the Ethics Committee of Kunshan Sixth People's Hospital (Approval No. KS202502012). This randomized controlled trial (RCT) was registered with the Primary Registry of the International Clinical Trial Registry Platform of the World Health Organization, under the “International Traditional Medicine Clinical Trial Registration Platform” (TTMCTR2025000447). Prior to participation, the purpose of the study was explained to all patients, and written informed consent was obtained.

### 2.2 Sample size

A pre-experimental approach was used to estimate the sample size in order to address limitations identified in previous studies. As a randomized controlled trial with a parallel-group design, the visual analogue scale (VAS) served as the primary continuous outcome variable. Sample size calculations were performed using G*Power 3.1.9.7 software, with a two-sided significance level of α = 0.05 and a power of 90% (1–β = 0.90). The minimum required sample size was determined to be 27 participants per group. Allowing for an anticipated dropout rate of 10%, at least 30 subjects were included in each group.

### 2.3 Participants

**Figure 1:**
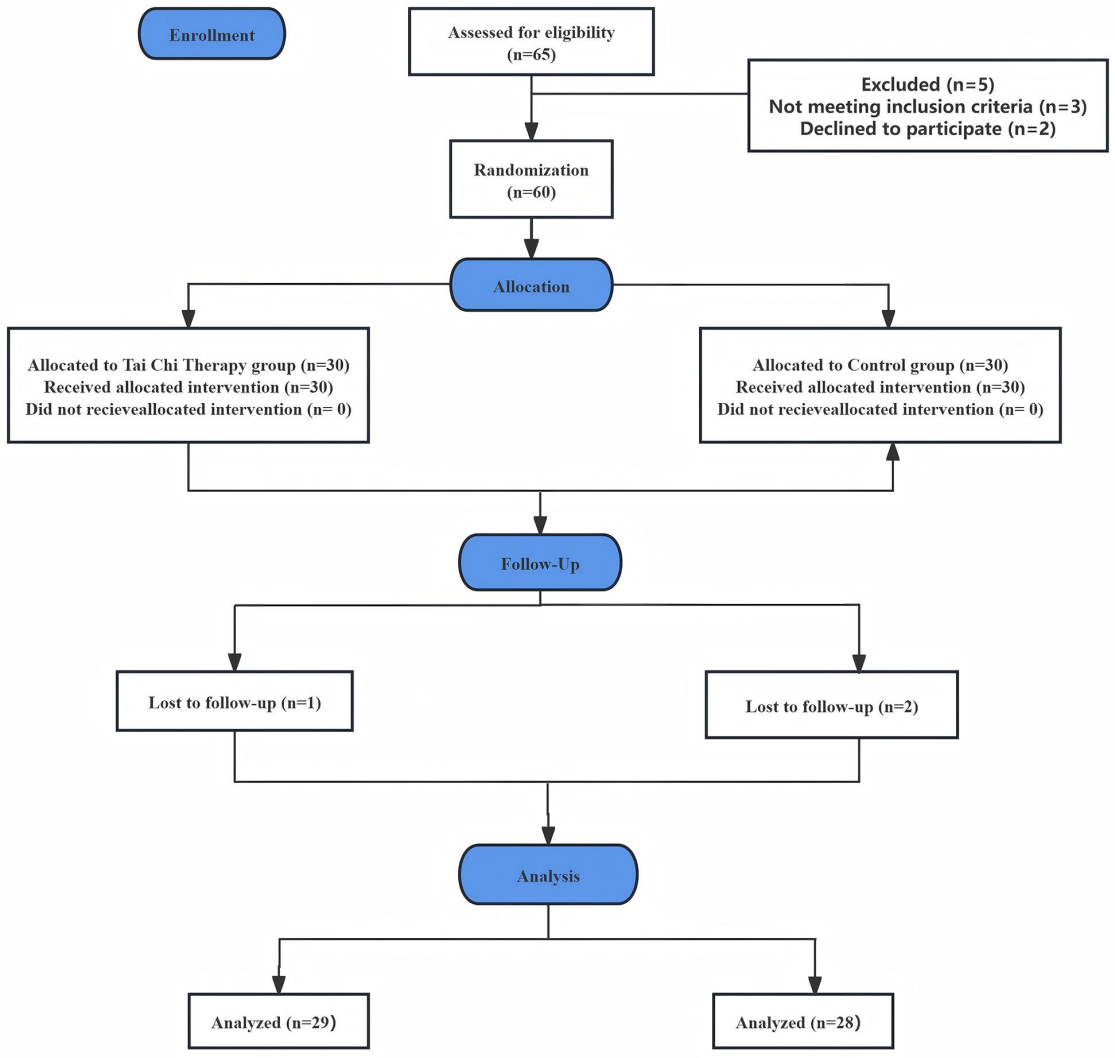
CONSORT flow chart for patient enrollment.

A total of 66 individuals were screened for eligibility, and 60 patients with CNSNP were ultimately recruited from the Department of Physical Therapy and Rehabilitation at Kunshan Sixth People's Hospital. All participants declined surgical intervention and provided written informed consent. The CONSORT flow diagram illustrating patient enrollment, allocation, follow-up, and analysis in this RCT is presented in Figure 1.

### 2.4 Inclusion criteria

1. Diagnosis consistent with the established criteria for CNNSP (15, 16), with persistent or recurrent pain lasting more than three months; 2. Age between 18 and 70 years; 3. No prior experience with Tai Chi; 4. Provision of written informed consent.

### 2.5 Exclusion criteria

(1). Presence of severe cardiovascular or cerebrovascular disease, arrhythmia, hematological disorders, malignancy, or significant organ dysfunction; (2). Menstruating or pregnant women; (3). Neck conditions such as nerve root compression, spinal cord compression, fracture, or infection; (4). Skin allergy or other serious dermatological conditions; (5). Local injection or surgical treatment within the previous three months; (6). Major psychiatric illness, cognitive impairment, or inability to cooperate with the study protocol; (7). Refusal to provide informed consent.

### 2.6 Elimination criteria

(1). Receipt of other treatment regimens, or withdrawal during the intervention or follow-up period; (2). Occurrence of severe adverse events necessitating a change in treatment.

### 2.7 Randomization and blinding

This study employed a randomized controlled trial design. An independent researcher generated a random allocation sequence using a random number table, assigning participants in a 1:1 ratio: lower values were allocated to the experimental group (EG), and higher values to the control group (CG). The EG received Tai Chi combined with intermediate frequency therapy, while the CG received intermediate frequency therapy alone. All interventions were administered by the same therapist to ensure consistency and standardization.

To minimize bias and ensure data authenticity, data collection was conducted by a separate researcher who was independent of the main study team and remained blinded to both group allocation and specific study hypotheses. This independent researcher had no direct communication with the principal investigator or the individual responsible for randomization, further reducing the potential for bias.

## 3 Outcome measures

All assessments were performed by a single, designated evaluator to minimize measurement error. Both groups were evaluated on the following parameters at baseline: VAS for pain, Neck Disability Index (NDI), D value of cervical physiological curvature (measured by x-ray), and cervical ROM score. At four weeks post-intervention, VAS, NDI, and cervical ROM scores were assessed. After eight weeks of intervention, VAS, NDI, D value of cervical physiological curvature, and cervical ROM scores were re-evaluated.

### 3.1 Primary outcome measures

The primary outcome was pain intensity, assessed using the VAS. The VAS consists of a 10 cm horizontal line marked with values from 0 to 10, where 0 indicates no pain and 10 represents the most severe pain imaginable. Patients were instructed to place a mark on the line corresponding to their perceived level of pain, and the resulting numerical value was recorded as the pain score.

### 3.2 Secondary outcome measures

The NDI was employed as the principal tool for evaluating self-reported disability in patients with neck pain. The NDI includes 10 items, each with six response options (scored 0–5), yielding a total possible score of 0 to 50. Higher scores reflect greater disability.

**Figure 2:**
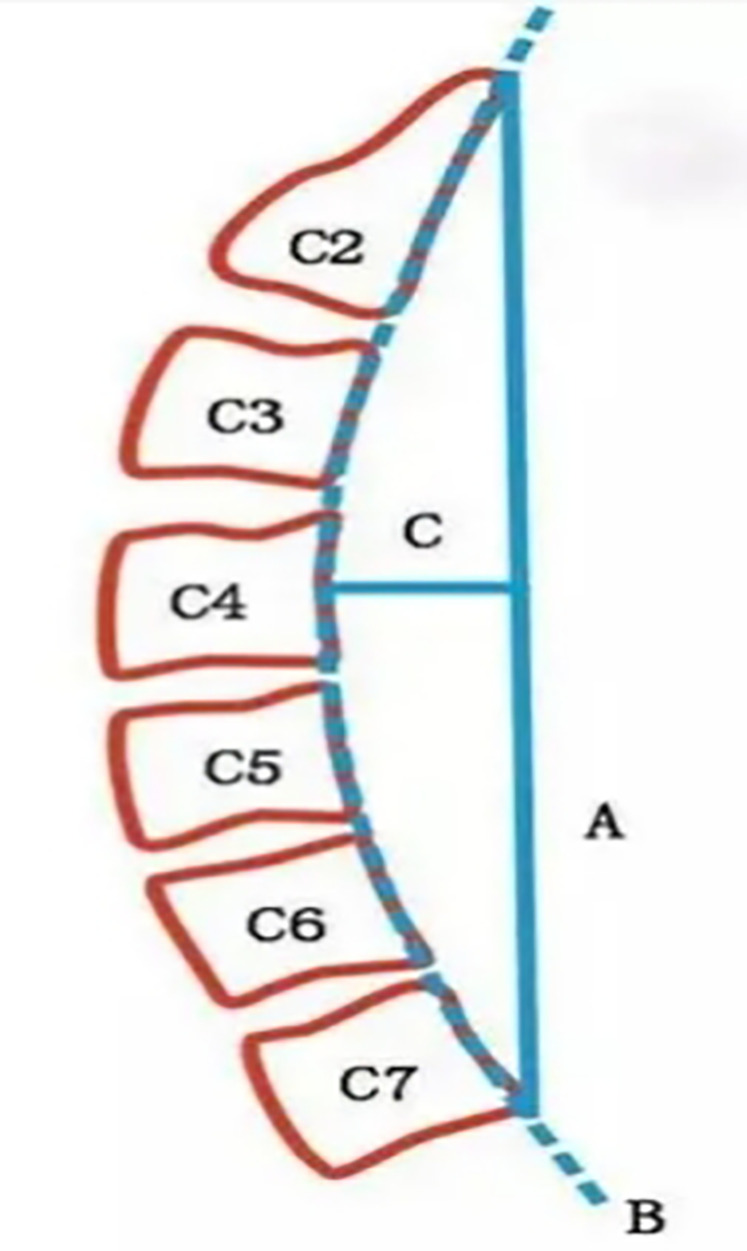
Borden's method diagram.

Cervical physiological curvature was assessed using x-ray imaging and the Borden method (17) (see Figure 2). This method involves drawing a straight line (A) from the posterior upper edge of the odontoid process of C2 to the posterior lower edge of the C7 vertebra. An arc (B) is traced along the posterior edges of each cervical vertebra. The maximum perpendicular distance (C) between lines A and B is measured as the cervical physiological curvature, denoted as the D value. A normal D value ranges from 7 to 17 mm; a D value < 7 mm indicates reduced cervical curvature, D = 0 mm denotes a straight cervical spine, and D < 0 mm indicates a reversed curvature.

Cervical ROM was measured using the F-JDCX goniometer (Changzhou Qianjing Rehabilitation Co., Ltd., China), assessing flexion, extension, lateral flexion, and rotation. ROM in each direction was quantified and scored as follows: Flexion, extension, or lateral flexion > 40°, and rotation > 75°: 3 points; Flexion, extension, or lateral flexion 30–40°, and rotation 60–75°: 2 points; Flexion, extension, or lateral flexion 20–29°, and rotation 45–59°: 1 point; Flexion, extension, or lateral flexion < 20°, and rotation < 45°: 0 points. The cumulative scores reflected the degree of cervical spine mobility for each participant.

## 4 Interventions

### 4.1 Experimental group

Prior to the commencement of the study, baseline demographic and clinical data—including age, gender, height, weight, and duration of CNSNP—were collected from all participants who met the inclusion criteria. All subjects completed pre-intervention assessments, including the VAS, NDI, D value of cervical curvature, and cervical ROM score.

Patients were subsequently treated in the rehabilitation department's outpatient clinic using the HB-ZP1 intermediate frequency interference electrotherapy device (Suzhou Haobo Medical Instrument Co., Ltd., Jiangsu Province, China; product standard number: YZB/Su 1508-2014). For therapy, three pairs of electrodes were positioned horizontally on the left, right, and posterior aspects of the participant's neck. Treatment was administered using prescription 02 (cervical spondylosis, stiff neck) on the device, with electrode output intensity gradually adjusted according to the patient's perception and tolerance. Each session lasted 20 min, administered once daily. Seven sessions comprised one treatment course, with a two-day rest between courses, for a total of two treatment courses.

Patients in the EG additionally received Tai Chi training, with the regimen as follows: 1. Training Frequency and Duration: Five sessions per week (four on weekdays, one on Saturday) for a total of eight weeks. 2. Session Structure: Each session included a 5 min warm-up, 30 min of Tai Chi practice, and 10 min of relaxation, totaling 45 min. 3. Safety Considerations: Participants were advised to avoid sudden, forceful neck rotations and to maintain proper alignment of the knees and toes during practice, ensuring knee flexion did not exceed the toes to prevent “Tai Chi knee”. Participants were also encouraged to avoid prolonged sitting or standing, as well as direct exposure of the neck to air conditioning or fans during the intervention period.

Supervision Protocol: Centralized Training: Conducted every Saturday from 7:00–9:00 a.m. in the rehabilitation medicine outpatient department, with a professional Tai Chi instructor leading the session. Attendance was mandatory for all EG participants, and sessions were video recorded. Home Training: At other times, instruction was provided via live video using Tencent Conference, and an EG WeChat group was established for training support. Participants were required to complete a training record form after each session and upload photos or videos of their practice to the group.

Participants were scheduled for follow-up visits at the hospital after four and eight weeks of intervention. At four weeks, VAS, NDI, and cervical ROM scores were assessed; at eight weeks, VAS, NDI, D value of cervical curvature, and cervical ROM scores were re-evaluated.

### 4.2 Control group

Baseline demographic and clinical characteristics—including age, sex, height, weight, and duration of pain—were collected from all CNSNP patients who met the inclusion criteria prior to the start of the study. Pre-intervention assessments, including VAS, NDI, D value, and cervical ROM scores, were also completed. In the control group, participants received only HB-ZP1 intermediate frequency interference electrotherapy, and all treatment courses, follow-up schedules, and related procedures were identical to those in the experimental group.

### 4.3 Statistical analysis

All statistical analyses were performed using SPSS version 23.0. Continuous variables conforming to a normal distribution were presented as mean ± standard deviation. The Shapiro–Wilk test was used to assess normality. For variables with a normal distribution, independent samples *t*-tests were employed for between-group comparisons; for non-normally distributed variables, the Mann–Whitney *U*-test was used for pairwise comparisons. Categorical variables were expressed as frequency and percentage, with group differences assessed using the chi-square test.

For repeated measures efficacy outcomes that satisfied normality and homogeneity of variance assumptions, the Mauchly's test of sphericity was first performed. If sphericity was confirmed, two-way analysis of variance (ANOVA) was used. If sphericity was violated, the Greenhouse-Geisser correction was applied. In the analysis of repeated measures ANOVA, if no interaction was observed between time and treatment factors, the main effect was assessed directly. If an interaction effect was present, further analysis was conducted: intra-group effects were assessed using one-way repeated measures ANOVA, and inter-group effects were analyzed by multivariate analysis of variance. A *p*-value < 0.05 was considered statistically significant, and *p* < 0.001 was interpreted as highly significant.

## 5 Results

### 5.1 Baseline characteristics

A total of 60 patients with CNSNP were initially enrolled in the study. During the trial, one participant in the EG was excluded after missing one week of Tai Chi intervention. Two participants in the CG discontinued: one due to a change in the original treatment plan, and another for personal reasons. Thus, 57 patients completed the trial, comprising 27 males and 30 females.

There were no statistically significant differences between the two groups in terms of age, gender, height, weight, or other baseline demographic and clinical characteristics (*p* > 0.05), confirming that the groups were comparable (see Table 1).

**Table 1:** Baseline characteristics (*n* = 57).

Features	CG, *n* = 28 (mean ± SD)	EG, *n* = 29 (mean ± SD)	*P*
Age (yrs)	36.50 ± 9.40	36.48 ± 9.56	0.995
Sex (M/F)	15/13	12/17	0.357
Height (cm)	169.57 ± 7.36	168.86 ± 7.20	0.715
Body weight (kg)	67.29 ± 8.79	67.59 ± 10.63	0.908
BMI (kg/m^2^)	23.38 ± 2.53	23.61 ± 2.87	0.743
Duration of pain (months), Median [IQR]	3.8 (4.25–8.00)	3 (4.00–7.00)	0.393

Mean, mean; SD, standard deviation; median: Median; [IQR], [interquartile range]; according to the normality test, continuous variables were expressed as Mean ± SD or Median [IQR]. Gender is a categorical variable, and N is used to represent the value. EG: Tai Chi combined with intermediate frequency treatment. CG: intermediate frequency therapy.

Following treatment, both groups demonstrated improvements in VAS, NDI, D value, and cervical ROM scores. Notably, the experimental group showed significantly greater improvement compared to the control group.

### 5.2 Main effect analysis of VAS scores during treatment

The VAS scores in both groups were normally distributed and satisfied the assumption of homogeneity of variance (*p* > 0.05). Mauchly's test of sphericity indicated that the sphericity assumption was violated (Mauchly's W = 0.772, *p* = 0.001), necessitating the application of the Greenhouse-Geisser correction.

Analysis of VAS scores revealed a statistically significant effect of time (*F* = 444.555, *p* < 0.001, *η*^2^ = 0.890), indicating that VAS scores changed significantly over the course of treatment in both groups. There was no significant main effect of group (*F* = 0.118, *p* = 0.733, *η*^2^ = 0.002), suggesting no overall difference in VAS scores between the two groups. However, a significant interaction was observed between time and group (*F* = 3.731, *p* = 0.036, *η*^2^ = 0.064), indicating that the change in VAS scores over time differed between treatment groups. Therefore, separate analyses were conducted to further examine the effects of time and group on VAS scores (see Table 2).

**Table 2:** VAS, NDI scores during treatment in both groups (*n* = 57).

Index	Peer group	Pretreatment baseline (mean ± SD)	Posttreatment (mean ± SD)		*F*, *P* (time)	*F*, *P* (between groups)	*F*, *P* (interaction)
			4 week	8 week			
VAS (score)	CG (*n* = 28)	5.429 ± 1.97	3.036 ± 1.45*	2.464 ± 1.48*^,#^	*F* = 444.555, *P* < 0.001	*F* = 0.118, *P* < 0.733	*F* = 3.731, *P* = 0.036
	EG (*n* = 29)	5.655 ± 1.97	2.690 ± 1.26*	2.172 ± 1.26*^,#^			
	T	0.434	−0.963	−0.805			
	P	0.666	0.340	0.425			
NDI (score)	CG (*n* = 28)	13.55 ± 6.39	7.14 ± 4.13*	5.14 ± 3.78*^,#^	*F* = 264.257,	*F* = 0.008,	*F* = 0.714,
	EG (*n* = 29)	13.64 ± 6.83	6.55 ± 4.79*	4.79 ± 3.01*^,#^			
	T	−0.052	−0.498	−0.388	*P* < 0.001	*P* < 0.733	*P* < 0.426
	P	0.959	0.620	0.699			

Both groups showed improvement in VAS and NDI.

* Indicates *p* < 0.05 compared to pretreatment.

^#^ Indicates *p* < 0.05 compared to 4 weeks posttreatment.

### 5.3 Simple effect analysis of VAS scores during treatment

The simple effect analysis indicated that there were no significant differences in VAS scores between the groups at baseline (*F* = 0.189, *p* = 0.666, *η*^2^ = 0.003), after 4 weeks of intervention (*F* = 0.927, *p* = 0.340, *η*^2^ = 0.017), or after 8 weeks of intervention (*F* = 0.647, *p* = 0.4215, *η*^2^ = 0.012).

However, the simple effect analysis for time showed significant within-group changes. In the Tai Chi combined with intermediate frequency therapy group, the effect of time was highly significant (*F* = 179.99, *p* < 0.001, *η*^2^ = 0.870). Similarly, in the intermediate frequency therapy group, the time effect was also significant (*F* = 123.548, *p* < 0.001, *η*^2^ = 0.821).

Multiple comparisons revealed that, in both the Tai Chi combined group and the intermediate frequency therapy group, VAS scores progressively decreased from pre-intervention to 4 weeks and further to 8 weeks post-intervention, with all comparisons reaching statistical significance (*p* < 0.001) (see Table 2).

### 5.4 Main effect analysis of NDI scores during treatment

NDI scores in both groups were normally distributed and met the homogeneity of variance assumption (*p* > 0.05). Mauchly's test of sphericity indicated a violation (Mauchly's W = 0.337, *p* < 0.001), and thus the Greenhouse-Geisser correction was applied.

Analysis showed a significant effect of time on NDI scores (*F* = 264.257, *p* < 0.001, partial *η*^2^ = 0.828), indicating substantial improvement in neck disability over the treatment period in both groups. There was no significant main effect of group (*F* = 0.008, *p* = 0.733, *η*^2^ = 0.002), nor was there a significant time-by-group interaction effect (*F* = 0.714, *p* = 0.426, *η*^2^ = 0.013) (see Table 2).

### 5.5 Comparison of x-ray cervical physiological curvature (D value) during treatment

After 8 weeks of intervention, both groups exhibited significant increases in the cervical physiological curvature D value compared to baseline (*p* < 0.05). Notably, the increase in D value was significantly greater in the EG than in the CG (*p* < 0.05). Specifically, patients in the EG showed a marked improvement in D value following 8 weeks of Tai Chi combined with intermediate frequency therapy. These findings suggest that the combined intervention was more effective in enhancing cervical physiological curvature than intermediate frequency therapy alone (see Table 3).

**Table 3:** D-value during treatment in both groups (*n* = 57).

Index	Peer group	Pretreatment Baseline (mean ± SD)	Posttreatment (mean ± SD)
			8 weeks
D(mm)	CG (*n* = 28)	0.43 ± 3.16	0.46 ± 3.11*
	EG (*n* = 29) 95% CI	0.83 ± 2.49 (−1.108, 1.906)	1.97 ± 2.26* (0.062, 2.940)
	T	0.531	2.091
	P	0.598	0.041

The EG showed greater improvement in D value than the CG.

* Indicates *p* < 0.05 compared to pretreatment.

### 5.6 Main effect analysis of cervical ROM parameters during treatment

The levels of cervical flexion ROM, extension ROM, bilateral rotation difference ROM, and bilateral lateral flexion difference ROM in both groups met the assumptions of normality and homogeneity of variance (*p* > 0.05). However, Mauchly's test of sphericity was not satisfied for any of these variables—flexion ROM (Mauchly's W = 0.657, *p* < 0.001), extension ROM (Mauchly's W = 0.636, *p* < 0.001), bilateral rotation difference ROM (Mauchly's W = 0.837, *p* = 0.008), and bilateral lateral flexion difference ROM (Mauchly's W = 0.809, *p* = 0.003) —therefore, the Greenhouse-Geisser correction was applied to all analyses.

Analysis revealed a statistically significant effect of time on both cervical flexion ROM (*F* = 198.623, *p* < 0.001, *η*^2^ = 0.783) and extension ROM (*F* = 326.128, *p* < 0.001, *η*^2^ = 0.856), indicating that these parameters changed significantly over the course of treatment in both groups. In contrast, there were no significant time effects for bilateral rotation difference ROM (*F* = 0.269, *p* = 0.731, *η*^2^ = 0.005) or bilateral lateral flexion difference ROM (*F* = 0.205, *p* = 0.776, *η*^2^ = 0.004).

No statistically significant group differences were found for cervical flexion ROM (*F* = 0.383, *p* = 0.539, *η*^2^ = 0.007), extension ROM (*F* = 3.934, *p* = 0.052, *η*^2^ = 0.067), bilateral rotation difference ROM (*F* = 0.681, *p* = 0.413, *η*^2^ = 0.012), or bilateral lateral flexion difference ROM (*F* = 2.618, *p* = 0.111, *η*^2^ = 0.045).

Furthermore, no significant interaction effects between time and group were observed for any of the cervical ROM measures: flexion ROM (*F* = 2.646, *p* = 0.092, *η*^2^ = 0.046), extension ROM (*F* = 0.645, *p* = 0.481, *η*^2^ = 0.012), bilateral rotation difference ROM (*F* = 1.003, *p* = 0.361, *η*^2^ = 0.018), or bilateral lateral flexion difference ROM (*F* = 2.336, *p* = 0.111, *η*^2^ = 0.041) (see Table 4).

**Table 4:** Cervical flexion and extension ROM during treatment in both groups (*n* = 57).

Index	Peer group	Pretreatment baseline (mean ± SD)	Posttreatment (mean ± SD)		*F*, *P* (time)	*F*, *P* (between groups)	*F*, *P* (interaction)
			4 week	8 week			
Flex (°)	CG (*n* = 28)	25.96 ± 7.32	32.04 ± 0.86*	38.79 ± 4.56*^,#^	*F* = 198.623, *P* < 0.001	*F* = 0.383, *P* = 0.539	*F* = 2.646, *P* = 0.092
	EG (*n* = 29)	23.03 ± 9.90	31.28 ± 0.78*	39.14 ± 6.89*^,#^			
	T	1.267	0.378	−0.227			
	P	0.211	0.707	0.822			
Extension (°)	CG (*n* = 28)	23.75 ± 6.50	29.93 ± 5.63*	36.43 ± 5.80*^,#^	*F* = 326.128, *P* < 0.001	F*F* = 3.934, *P* = 0.052	*F* = 0.645, *P* = 0.481
	EG (*n* = 29)	20.07 ± 8.23	27.31 ± 6.75*	32.93 ± 6.21*^,#^			
	T	1.870	1.587	2.196			
	P	0.067	0.118	0.032			
Bilateral rotation difference ROM (°)	CG (*n* = 28)	1.50 ± 7.94	2.82 ± 4.91	2.43 ± 3.86	*F* = 0.269, *P* = 0.731	*F* = 0.681, *P* = 0.413	*F* = 1.003, *P* = 0.361
	EG (*n* = 29)	1.72 ± 5.68	1.35 ± 4.42	0.90 ± 3.44			
	T	−1.23	1.195	1.583			
	P	0.903	0.237	0.119			
Bilateral lateral flexion difference ROM (°)	CG (*n* = 28)	1.36 ± 3.32	1.89 ± 3.24	1.43 ± 2.54	*F* = 0.205, *P* = 0.776	*F* = 2.618, *P* = 0.111	*F* = 2.336, *P* = 0.111
	EG (*n* = 29)	0.76 ± 3.71	−0.21 ± 3.65	0.35 ± 3.31			
	T	0.641	2.296	1.383			
	P	0.524	0.026	0.172			
ROM integral	CG (*n* = 28)	1.46 ± 0.58	1.11 ± 0.32*	0.64 ± 0.49*^,#^	*F* = 112.990, *P* < 0.001	*F* = 1.753, *P* = 0.191	*F* = 3.737, *P* = 0.030
	EG (*n* = 29)	1.45 ± 0.57	1.07 ± 0.26*	0.31 ± 0.47*^,#^			
	T	0.105	0.501	2.618			
	P	0.917	0.618	0.011			

Both groups showed improvement in ROM.

* Indicates *p* < 0.05 compared to pretreatment.

^#^ Indicates *p* < 0.05 compared to 4 weeks posttreatment.

### 5.7 Main effect analysis of cervical spine ROM scores during treatment

The cervical flexion ROM scores for both groups were normally distributed and met the homogeneity of variance assumption (*p* > 0.05). Mauchly's test of sphericity was satisfied (Mauchly's W = 0.996, *p* = 0.909), indicating that sphericity was maintained; thus, Greenhouse-Geisser correction was not required.

Analysis of cervical spine ROM scores revealed a statistically significant effect of time (*F* = 112.990, *p* < 0.001, partial *η*^2^ = 0.807), indicating that ROM scores changed significantly over the course of treatment in both groups. There was no significant main effect of group (*F* = 1.753, *p* = 0.191, *η*^2^ = 0.031), suggesting that overall ROM scores did not differ significantly between groups. However, a significant interaction effect between time and group was observed (*F* = 3.737, *p* = 0.030, *η*^2^ = 0.122), indicating that the pattern of change in ROM scores over time varied according to the treatment method. Consequently, separate analyses were conducted to further examine time and intergroup effects (see Table 4).

### 5.8 Simple effect analysis of cervical spine ROM scores during treatment

The simple effect analysis indicated that there were no significant differences in cervical spine ROM scores between the two groups at baseline (*F* = 0.011, *p* = 0.917, *η*^2^ = 0.000) or after 4 weeks of intervention (*F* = 0.252, *p* = 0.618, *η*^2^ = 0.005). However, after 8 weeks of intervention, the difference between groups became significant (*F* = 6.856, *p* = 0.011, *η*^2^ = 0.111).

Within-group analysis revealed that the effect of time was significant in both the Tai Chi combined with intermediate frequency therapy group (*F* = 79.255, *p* < 0.001, *η*^2^ = 0.586) and the intermediate frequency therapy group alone (*F* = 38.192, *p* < 0.001, *η*^2^ = 0.586).

Multiple comparisons demonstrated that, in both groups, ROM scores significantly decreased from pre-intervention to 4 weeks, and further to 8 weeks post-intervention (all *p* < 0.05). This indicates a significant improvement in cervical ROM over the course of treatment in both groups, with a greater effect observed in the Tai Chi combined group at 8 weeks (see Table 4).

## 6 Discussion

### 6.1 The effect of Tai Chi on pain in patients with CNSNP

Tai Chi is a traditional mind-body practice that integrates meditation with slow, gentle, and fluid movements. It is widely used for the management of chronic pain and has been shown to effectively reduce pain intensity in patients with CNSNP (18). In the present study, VAS scores in the experimental group were significantly lower after both 4 and 8 weeks of treatment compared to baseline (*p* < 0.001). Moreover, VAS scores further decreased at 8 weeks compared to the 4-week assessment (*p* < 0.001), indicating a progressive pain reduction over the course of the intervention. These findings suggest that Tai Chi combined with intermediate frequency therapy can significantly improve pain perception in CNSNP patients. This result aligns with several randomized controlled trials demonstrating the analgesic effects of Tai Chi in chronic pain populations (19–21).

The mechanisms by which Tai Chi alleviates pain may be attributed to several factors. First, Tai Chi movements can activate muscle spindle receptors by stretching muscles and mobilizing joints, thereby increasing the neuronal activity of type Ia and type II sensory fibers, activating the gate control system in the spinal cord, and inhibiting the transmission of pain signals via type C fibers, resulting in pain relief (22). Second, Tai Chi practice is thought to promote autonomic balance by suppressing sympathetic nervous activity and enhancing parasympathetic activity (23), thereby reducing chronic pain and inhibiting inflammation (24). Third, neuroimaging studies have shown that individuals who engage in long-term Tai Chi practice exhibit reduced functional homogeneity in the left anterior cingulate cortex and increased cortical thickness in the inferior insular sulcus (25). These alterations in brain structure and function may contribute to enhanced pain relief. Importantly, Tai Chi is a safe, low-cost intervention that can be practiced at home without the need for specialized equipment, making it particularly accessible for the CNSNP population (18).

### 6.2 The effect of NDI on pain in patients with CNSNP

The NDI is a widely used assessment tool that reflects neck pain and associated symptoms, objectively evaluating cervical spine function and the impact on activities of daily living. It also provides insight into patients’ perception of treatment efficacy following intervention. In this study, NDI scores in the experimental group were significantly lower after both 4 and 8 weeks of treatment compared to baseline (*p* < 0.001). Furthermore, NDI scores continued to decrease significantly from 4 to 8 weeks post-intervention (*p* < 0.001). These results indicate that Tai Chi combined with intermediate frequency therapy effectively improves neck symptoms and functional impairment in patients with CNSNP. This finding is consistent with previous research, which has also demonstrated the beneficial effects of Tai Chi in reducing neck symptoms and improving cervical dysfunction in this population (18).

The mechanisms underlying the improvement in NDI scores with Tai Chi intervention may be multifaceted. Tai Chi exercises target the neck muscle groups, enhancing both strength and endurance, which helps maintain dynamic stability of the cervical spine and reduces pain resulting from muscle fatigue and imbalance (26). Sleep disturbance is a common comorbidity in patients with neck pain and is included as an index on the NDI scale. Tai Chi practice, with its emphasis on regulating body, breath, and mind, can alleviate anxiety and other negative emotions in CNSNP patients. It has also been shown to reduce levels of pro-inflammatory cytokines associated with sleep disturbances, such as tumour necrosis factor-α (TNF-α) and interleukin-1β (IL-1β), thereby improving sleep quality (27). Additionally, the NDI includes assessment of concentration, and there is evidence that α brainwave activity is closely related to attentional control (28). After relaxation through Tai Chi practice, the α2 rhythm in the parietal and occipital lobes significantly increases compared to pre-relaxation levels (29). This suggests that enhanced attention control during Tai Chi may be associated with the modulation of cortical α-wave activity.

### 6.3 The effect of cervical physiological curvature on pain in patients with CNSNP

The physiological curvature of the cervical spine is a naturally formed, forward convex structure critical for maintaining the overall mechanical balance of the spine. In the present study, after 8 weeks of intervention, the D value of cervical curvature in the EG increased significantly compared to baseline (*p* < 0.05), and was also significantly higher than that of the CG at the end of treatment (*p* = 0.041). These findings indicate that Tai Chi combined with intermediate frequency therapy is more effective than intermediate frequency therapy alone in improving cervical physiological curvature in patients with CNSNP. This result is consistent with several randomized controlled trials that have shown beneficial effects of Tai Chi on cervical curvature in individuals with cervical spondylosis (14, 30).

Prolonged static positioning or sustained flexion of the cervical spine increases the preload on both cervical flexor and extensor muscles, leading to continuous tonic contraction. This persistent contraction can result in muscle fiber injury and decreased muscle strength, ultimately disrupting the dynamic and static balance of the cervical spine, compromising its mechanical properties, and predisposing to degenerative changes (31). Tai Chi practice applies static tension in multiple directions—upward, downward, left, and right—allowing for natural relaxation of the cervical and entire spinal column, elongation of cervical muscle ligaments, and reduction of abnormal stress on the neck and shoulder muscles. This facilitates restoration of static and dynamic balance in musculoskeletal joints, correction of joint force lines, and enhanced joint stability, thereby helping to maintain the physiological curvature of the cervical and thoracic spine (32). Moreover, long-term adherence to Tai Chi not only delays the progression of cervical degenerative disease but can also improve existing degenerative changes (31). For example, Liu et al. (14) demonstrated that six months of Chen-style Tai Chi combined with physical rehabilitation not only significantly corrected the anterior curvature of the cervical spine but also improved lumbar curvature and increased vital capacity.

### 6.4 The effect of ROM on pain in patients with CNSNP

Prolonged neck flexion and physical inactivity can lead to muscle adhesions and stiffness around the spine, decreased ligament extensibility, reduced physiological curvature, and various degrees of pain. Joint mobility is a critical clinical indicator for assessing and evaluating joint function, and is especially significant in the context of cervical spine disorders. Patients with neck pain typically experience restricted cervical ROM due to pain and associated dysfunction.

In this study, cervical flexion and extension ROM in the EG improved significantly after both 4 and 8 weeks of treatment compared to baseline (*p* < 0.001). Notably, there were further significant increases in cervical flexion and extension ROM from week 4 to week 8 (*p* < 0.001). Additionally, compared with the CG, EG demonstrated a significantly greater reduction in the bilateral flexion ROM difference after 4 weeks (*p* = 0.026) and a significantly higher overall ROM score after 8 weeks of treatment (*p* = 0.011), indicating substantial improvements in cervical mobility. These findings suggest that Tai Chi combined with intermediate frequency therapy is more effective than intermediate frequency therapy alone in improving cervical ROM in patients with CNSNP. This superior effect may be attributable to Tai Chi's capacity to enhance both stability and flexibility in the joints and trunk.

A major advantage of Tai Chi as an active therapeutic exercise is its ability to fully engage patients in their clinical care, fostering subjective initiative and psychological positivity. Regular and structured Tai Chi practice can help eliminate or alleviate pathological conditions through movement, ultimately restoring normal physiological function in the cervical joints (33). Moreover, Tai Chi has been shown to enhance the elasticity and flexibility of periarticular connective tissue, increase the strength and endurance of neck muscles, improve the tensile strength of ligaments and joint capsules, and promote blood circulation, all of which contribute to increased cervical ROM in patients with CNSNP (18, 34). These findings are consistent with the present study. Furthermore, the twisting and rotational movements of the head inherent in Tai Chi postures may stretch key muscles, such as the cervical trapezius and platysma, thereby expanding the range of motion of the neck. With improved muscle flexibility and strength, the toughness of the neck musculature is enhanced, leading to improved joint mobility.

In summary, Tai Chi can be incorporated into rehabilitation programs for patients with CNSNP through its clinical application in alleviating pain, improving the NDI score, restoring cervical lordosis, and enhancing ROM.

## 7 Conclusion

For patients with CNSNP, the combination of Tai Chi and intermediate frequency therapy has been shown to effectively alleviate pain, enhance neck function, and improve ROM. Compared to intermediate frequency therapy alone, this combined approach significantly improves the physiological curvature of the cervical spine in individuals with CNSNP. Furthermore, these findings suggest that Tai Chi is an effective and safe therapeutic option, and may represent a promising alternative for the management of CNSNP.

## 8 Innovation

This study presents several key innovations. First, it introduces Tai Chi as a promising new intervention for the treatment of CNSNP, potentially offering a novel direction for conservative management of this condition. Second, the use of x-ray-based cervical physiological curvature measurements and cervical ROM assessments provides objective and quantifiable indicators for evaluating treatment efficacy in CNSNP. The results of this study confirm that Tai Chi is a safe and effective conservative therapy for patients with CNSNP.

## 9 Limitations

However, several limitations should be acknowledged. Due to constraints in researcher experience, time, and funding, the study has notable shortcomings. The sample size was relatively small, and long-term follow-up was not conducted, which may limit the generalizability of the findings. In addition, out of consideration for patients' economic burden and concerns regarding radiation exposure, cervical lateral x-ray examinations were only performed before and after the intervention (8 weeks post-treatment), rather than at multiple time points. Lastly, biomechanical research on its effects has focused primarily on the lower limbs, knee joints, and plantar regions. There remains a paucity of studies investigating its biomechanical impact on the head, neck, upper limbs, and spine. Future research should expand the mechanical analysis of Tai Chi interventions to these regions to further elucidate its therapeutic mechanisms. Despite these limitations, the study provides a valuable foundation for future trials.

## Data Availability

The raw data supporting the conclusions of this article will be made available by the authors, without undue reservation.
